# MHCII Expression on Peripheral Blood Monocytes in Canine Lymphoma: An Impact of Glucocorticoids

**DOI:** 10.3390/ani12162135

**Published:** 2022-08-19

**Authors:** Alicja Rzepecka, Dariusz Jagielski, Anna Cywińska, Rafał Sapierzyński, Magdalena Żmigrodzka, Olga Witkowska-Piłaszewicz, Anna Winnicka

**Affiliations:** 1 Institute of Veterinary Medicine, Warsaw University of Life Sciences, 02-787 Warsaw, Poland; 2 Veterinary Surgery “Białobrzeska”, 02-344 Warsaw, Poland; 3 Faculty of Biological and Veterinary Sciences, Nicolaus Copernicus University, 87-100 Toruń, Poland

**Keywords:** canine lymphoma, flow cytometry, glucocorticoids, immunosuppression, monocytes

## Abstract

**Simple Summary:**

Loss or decreased expression of human leukocyte antigen—D-related (HLA-DR) on the surface of monocytes is related to the dysfunction of the immune system and was reported in human neoplasia, including lymphoma. Canine lymphoma is frequently presented as a valuable comparative model for studies on human non-Hodgkin’s lymphoma. However, there are no studies on the expression of analogue proteins—MHCII antigens—on monocytes in canine lymphoma. In this study, we have evaluated the changes in the expression of MHCII on monocytes in the blood of dogs with lymphoma before any treatment and in dogs that had previously received glucocorticoids. Glucocorticoids are often used by clinicians as first drugs after diagnosis for immediate health improvement and are known to impact monocyte number. We have shown an increase in the percentage of MHCII− monocytes, regardless of treatment. However, only in dogs that had received glucocorticoids were changes in the proportion of MHCII+ and MHCII− monocytes reflected also by the changes in the number of MHCII− monocytes in the blood, which was significantly higher. Evaluating the changes in canine monocytes might be helpful in the diagnosis of various tumor types, monitoring of the treatment or assessing the immune status of dogs.

**Abstract:**

An increase in the percentage of monocytes with reduced HLA-DR expression and immunosuppressive properties has been reported in numerous human neoplastic diseases, including lymphoma. However, there are no analogous studies on phenotypical variations in the peripheral blood monocytes in dogs with lymphoma. The aim of this study was to determine the difference in the expression of the MHCII molecule on peripheral blood monocytes in dogs with lymphoma before any treatment (NRG) and in dogs that had previously received glucocorticoids (RG) in comparison to healthy dogs. Flow cytometry immunophenotyping of peripheral blood leukocytes was performed using canine-specific or cross-reactive antibodies against CD11b, CD14 and MHCII. In the blood of dogs with lymphoma (NRG and RG), compared to that of healthy ones, the MHCII+ and MHCII− monocytes ratio was changed due to an increase in the percentage of MHCII− monocytes. The number of MHCII− monocytes was significantly higher only in RG dogs compared to healthy ones, which might result from the release of these cells from the blood marginal pool due to the action of glucocorticoids. Our results encourage further studies to assess if changes in MHCII expression affect immune status in dogs with lymphoma.

## 1. Introduction

The constitutive presence of major histocompatibility antigen class II (MHCII) proteins is assigned to professional antigen-presenting cells: dendritic cells, B lymphocytes, monocytes and macrophages. Their main role is to present exogenous antigens to T helper cells, which leads to the activation of a specific immune response [1]. Decreased expression of MHCII on peripheral blood monocytes has been often discussed in the context of various diseases. Significant loss of human leukocyte antigen—D-related (HLA-DR) on the surface of monocytes has been observed during septic shock and in severely burned patients with sepsis [2,3]. These changes are related to the dysfunction of the immune system [4]. Similar observation has been made in dogs with septic and nonseptic inflammatory diseases and in multiple organ dysfunction syndrome [5]. Major histocompatibility complex (MHC) antigens also participate in the anti-tumor response [6]. Tumor cell antigens are processed and presented in the context both MHC class I and MHC class II proteins, thus being a potential target for CD8+ T lymphocytes but also CD4+ T cells with cytotoxic activity [7,8,9].

The increase in the percentage of monocytes with no or reduced HLA-DR expression has also been reported in human neoplasia, including lymphoma [10,11,12]. Moreover, these studies indicated the immunosuppressive nature of these cells. Lin et al. have observed that monocytes in non-treated patients with non-Hodgkin’s lymphoma (NHL) had significantly reduced expression of HLA-DR and CD120b [11]. Increased percentage of these cells correlated with aggressiveness of the disease and impairment of immune function [11]. The authors have also shown that CD14+HLA-DRlow/− monocytes inhibited lymphocyte proliferation and IFN-γ production, presumably due to their interference in the arginine metabolism. Comparative analysis among NHL types indicated the highest percentage of CD14+HLA-DRlow/− monocytes in patients with diffuse large B-cell lymphoma (DLBCL) comparing to follicular or indolent lymphoma. Xiu et al., have reported that malignant B-cells produced IL-10 in NHL patients thus promoted the decrease in MHCII expression on monocytes and the increase in the percentage of CD14+HLA-DRlow/− cells [13].

Canine lymphoma, especially DLBCL, is often presented as a valuable comparative model for the studies on human NHL due to many similarities they share, including clinical, histological and cytogenetic features [14,15,16,17]. However, human studies are still more informative than those conducted in companion animals and there is no similar data on MHCII expression on monocytes in canine lymphoma.

Most lymphoma treatment protocols in canine patients include glucocorticoids. In some cases, e.g., lack of consent of the owners (high costs of treatment, cytotoxicity, the need for special care over the animal) or lack of availability of cytostatics, glucocorticoids may be used alone as palliative therapy, which is often practiced [18]. Used as single-agent therapy they may lead to the extension of the patient’s lifespan, but less effectively than in combination with cytostatics [19]. The efficacy of glucocorticoids in the treatment of lymphomas is based on lymphotoxicity; however, the adverse reactions may, in the long run, contribute to the deterioration of the patient’s condition (e.g., weight loss due to increased catabolism of skeletal muscles) [19]. Interestingly, the treatment of dogs with glucocorticoids before the introduction of chemotherapy, negatively correlates with overall survival time and makes neoplastic cells less amenable to treatment with chemotherapeutics [20].

In this study we verified the hypothesis about the differences in the expression of MHCII molecule on the peripheral blood monocytes in dogs with lymphoma that previously had or had not received glucocorticoids, comparing them to healthy individuals.

## 2. Materials and Methods

### 2.1. Dogs and Sample Collection

Peripheral blood samples of dogs newly diagnosed with lymphoma (*n* = 18) and healthy controls (*n* = 11) were collected into tubes with dipotassium ethylenediaminetetraacetic acid (K2-EDTA). Samples were kept at a temperature of 4 °C before flow cytometry analysis, (performed within 18 h from sampling). The inclusion criteria for the healthy control group were: no signs or history of neoplastic disease; lack of clinical and laboratory signs of the disease; and lack of treatment or vaccination two weeks before sampling. Blood samples from dogs with lymphoma were collected before introduction of any anti-cancer treatment with chemotherapeutic drugs. According to clinical records, eight dogs with lymphoma had previously received one or more doses of glucocorticoids (Table S1). As glucocorticoids are known to change the number of monocytes in canine blood, the animals were grouped as dogs with lymphoma receiving glucocorticoids (RG, *n* = 8) and dogs with lymphoma without glucocorticoid treatment (NRG, *n* = 10). The blood collection covered non-experimental routine procedures based on a veterinary license consented by the dogs’ owners. The material used in the study was an excess blood (1–2 mL) collected for routine diagnostic examination. Therefore, according to the European directive EU/2010/63 and local regulations regarding animal experiments, there was no need for the approval of Ethical Committee.

The control group consisted of healthy dogs aged from 1 to 14 years (median—6 years), four males (one castrated) and seven females (four castrated). There were two mixed-breed dogs, two Miniature Schnauzers, one Belgian Malinois Sheepdog, one Cavalier King Charles Spaniel, three Boxers and two Border Collies. Dogs with lymphoma were between 4 and 15 years old (median—7 years 9 months). Of these, there were 12 males (4 castrated) and 6 females (3 castrated). There were seven mixed-breed dogs, one Rottweiler, one Golden Retriever, one Doberman, one German Shepherd, one Caucasian Shepherd Dog, one Boxer, one Bouvier des Flandres, one English Cocker Spaniel, one Siberian Husky and two Bernese Mountain Dogs.

The clinical stage of lymphoma was determined based on medical data review (clinical symptoms, physical examination, blood morphology and hematological examination and ultrasonographic imaging) using common classification criteria for multicentric lymphoma [18]. All dogs with suspected lymphoma presented generalized enlargement of peripheral lymph nodes. In two dogs (2/18), the symptoms were limited only to enlarged peripheral lymph nodes—classified into stage III. Spleen and abdominal nodes were involved in 83% cases (15/18) (stage IV). Based on clinical presentation and blood testing, bone marrow aspirates were obtained only from one dog, allowing its classification into stage V. In summary, all patients represented multicentric lymphoma of stage III or greater. The diagnosis of lymphoma was made by cytology followed by immunocytochemistry and flow cytometric analysis performed in the Department of Veterinary Pathology and Diagnostics, Institute of Veterinary Medicine, Warsaw University of Life Sciences. B-cell lymphoma (CD21+CD79a+) was found in 14 cases. According to Kiel classification, 2 dogs were diagnosed with lymphoblastic B-cell lymphoma (Rottweiler and mixed-breed dog, both RG) and 12 with centroblastic polymorphic B-cell lymphoma (morphologically corresponded to DLBCL). Four dogs were diagnosed with pleomorphic mixed small and large T cell lymphoma (CD3+CD4+; Boxer and mixed-breed dog NRG, English Cocker Spaniel and mixed-breed dog RG).

### 2.2. Flow Cytometry

For flow cytometry immunophenotyping, an appropriate volume of peripheral blood was aliquoted to obtain 1 × 10^6^ leukocytes per tube. To assess the cellularity of the samples, total white blood cell count was determined using an automated hematology analyzer (ABX Horiba, Kyoto, Japan). Samples were labeled with the following panel of monoclonal antibodies: rat anti-dog CD45 APC-conjugated (clone YKIX716.13, Bio-Rad, Hercules, CA, USA), mouse anti-dog CD11b unconjugated (clone CA16.3E10, Bio-Rad), rat anti-dog MHC class II FITC-conjugated (clone YKIX334.2; Bio-Rad) and mouse anti-human CD14 FITC or Alexa Fluor^®^647-conjugated (clone TÜK4; Bio-Rad) that cross-reacts with canine CD14 molecules [21]. Controls included isotype controls or omission of the primary antibody. Specimens were incubated at 4 °C for 30 min in the dark, followed by red blood cell lysis (15 min, room temperature, in the dark; lysing solution BD FACS^TM^). Next, cells were washed twice (5 min, 300× *g*) in 500 μL of phosphate-buffered saline (Sigma-Aldrich, Hamburg, Germany) prior to cytometric analysis. Primary antibodies were added into tubes in the following combinations: I CD45:APC/CD14:FITC, II CD11b/CD14:AlexaFluor^®^647/MHC class II:FITC. All antibodies were directly conjugated to fluorochromes, except for CD11b, which was immunolabeled with a secondary phycoerythrin-conjugated antibody (PE rat anti-mouse IgG1; BD Pharmingen). After the addition of the secondary antibody, the cells were incubated (30 min, 4 °C, in the dark) and washed twice (5 min, 300× *g*). Appropriate concentration of each antibody was previously determined in order to obtain optimal staining results.

**Figure 1:**
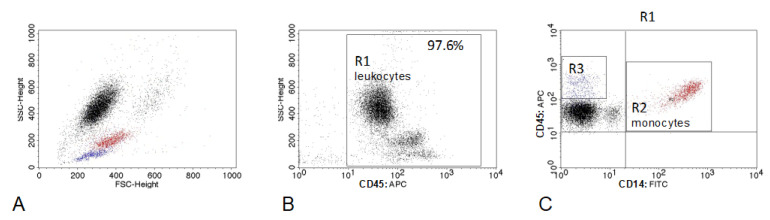
Representative dot plots depicting the gating strategy of canine peripheral blood leukocytes. (**A**) Forward scatter (FSC-hight) vs. side scatter (SSC-hight) dot plot showing leukocyte populations (population colors result from the regions set on the cytogram C); (**B**) debris and non-cellular elements were eliminated by setting a region defining CD45+ leukocytes (R1) on the SSC vs. CD45:APC dot plot; (**C**) leukocyte populations “(R1)” were analyzed on the CD45:APC/CD14:FITC dot plot in order to separate CD14+ monocytes (R2—red color) from lymphocytes (R3—blue color).

**Figure 2:**
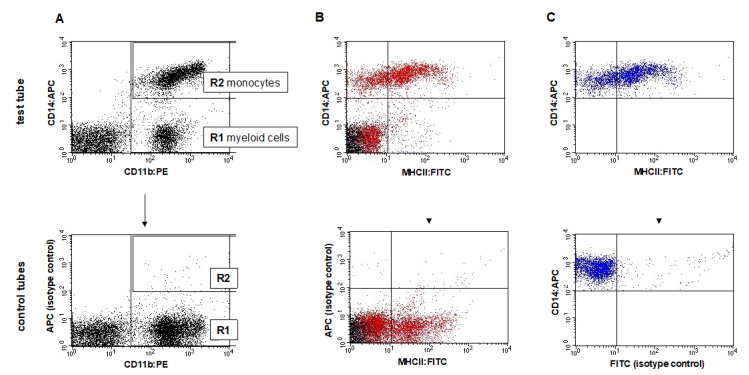
Representative dot plots depicting gating strategy of peripheral blood monocytes of dog with lymphoma. (**A**) Regions were set for all CD11b+ myeloid cells (R1) and for CD14+ monocytes (R2) on the CD14:APC vs. CD11b:PE double fluorescence dot plot; (**B**) distribution of all leukocytes (CD11b+ cells—red color) and (**C**) monocytes (from the R2 region—blue color) according to the expression of CD14 and MHCII. Bottom-row dot plots show isotype controls, separate for each Ab: anti-CD14 (**A**,**B**) and anti-MHCII (**C**). Controls allowed us to set the quadrants.

Cells were analyzed using a FACSCalibur cytometer and CellQuest software (Becton Dickinson, Franklin Lakes, NJ, USA). The analysis was performed immediately after staining. A total of 10,000 cells were counted from each sample. First, the location of particular leukocyte populations was determined using anti-CD45 and -CD14 antibodies (“leukogate”). The percentage of CD45+ cells for each sample was also calculated to exclude elements of non-leukocyte origin and debris. It was also determined that all CD14+ monocytes express CD45, which allowed us to assess the percentage of CD14+ monocytes within CD45+ cell population and next, their count. The gating strategy of cells labeled with CD45:APC and CD14:FITC antibodies is shown in Figure 1. In the next step, the expression of MHCII molecule on CD11b+CD14+ monocytes was analyzed by setting gates on the basis of isotype controls and unlabeled cells (negative control). Exemplary dot plots showing the cell analysis method and gating strategy are presented in Figure 2.

The number of monocytes and their individual subpopulations were determined based on the white blood cell count obtained from the automated analyzer and the percentage of CD14+ monocytes relative to all leukocytes (CD45+ cells).

### 2.3. Statistical Analysis

Statistical analysis was performed using GraphPad Prism 6.0 software (San Diego, CA, USA). The results are presented as the arithmetic mean ± standard deviation. To determine the significance of differences among mean values in the three groups, the non-parametric Kruskal–Wallis test and with post-hoc analysis (Dunn’s test for multiple comparisons) were used. For all analysis, the value of *p* < 0.05 was considered significant.

## 3. Results

**Figure 3:**
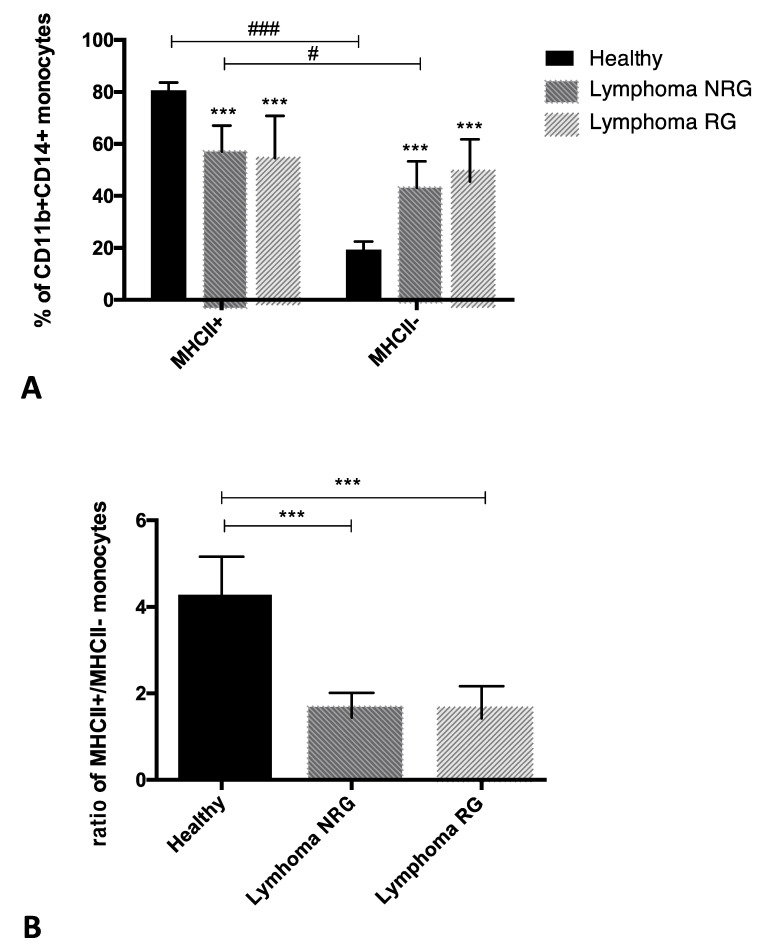
(**A**) The percentage of CD11b+CD14+ monocytes: MHCII+ and MHCII−, in the peripheral blood of healthy dogs (*n* = 11) and dogs with lymphoma: not receiving any drugs (NRG, *n* = 10) and receiving glucocorticoids (RG, *n* = 8). Arithmetic mean ± standard deviation. Significant differences between the percentage of MHCII+ and MHCII− monocytes among each group: # *p* < 0.05, ### *p* ≤ 0.001 (Wilcoxon) and for the percentage of MHCII+ or MHCII− monocytes in comparison to healthy dogs: *** *p* ≤ 0.001 (no significant differences between dogs with lymphoma NRG and RG) (Kruskal–Wallis and post hoc Dunn analysis). (**B**) The results are shown as the ratio of CD11b+CD14+ monocyte percentages: MHCII+ to MHCII−. Arithmetic mean ± standard deviation. Significant differences between groups: *** *p* ≤ 0.001 (Kruskal–Wallis and post hoc Dunn analysis).

**Figure 4:**
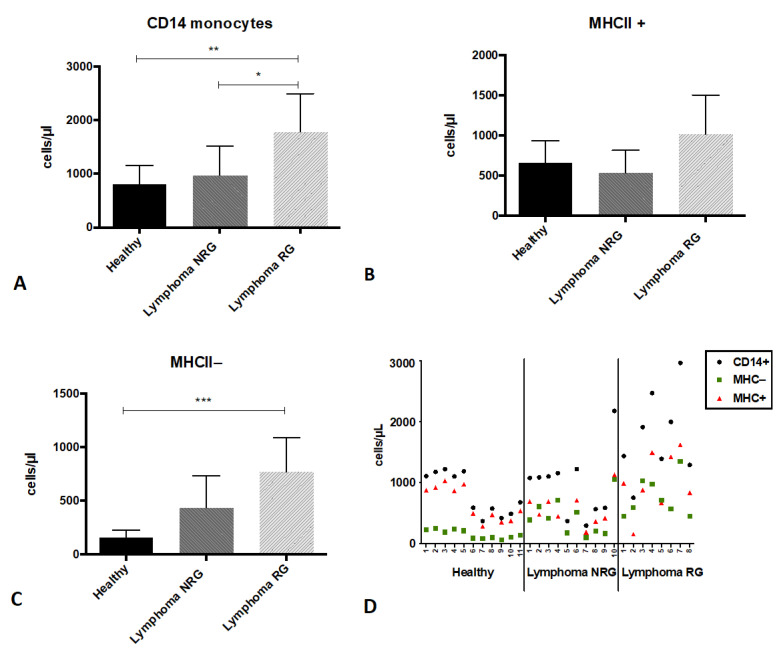
The number of CD14+ monocytes in the peripheral blood of healthy dogs (*n* = 11) and dogs with lymphoma: not receiving any drugs (NRG, *n* = 10) and receiving glucocorticoids (RG, *n* = 8). (**A**) Total number of CD14+ monocytes per microliter of peripheral blood. The number of CD14+ cells was calculated based on their percentage in relation to all leukocytes (CD45+ cells) and white blood cell count. (**B**) The number of MHCII+ and (**C**) MHCII− monocyte subsets. Arithmetic mean ± standard deviation. Significant differences between groups: * *p* < 0.05, ** *p* ≤ 0.01, *** *p* ≤ 0.001 (Kruskal–Wallis and Dunn post hoc analysis). (**D**) The numbers of all evaluated cell populations in individual patients: MHCII− and MHCII+ monocytes as well as all CD14+ cell population.

Cytometric analysis of MHCII expression on CD11b+CD14+ monocytes in the peripheral blood of dogs allowed us to distinguish two subpopulations of these cells: MHCII-positive (MHCII+) and MHCII-negative (MHCII−). The differences in the percentage of MHCII+ and MHCII− monocytes within and between groups are shown in Figure 3A. The percentage of MHCII− monocytes in comparison to MHCII+ cells was significantly lower in healthy dogs (19.40% ± 3.03 vs. 80.60% ± 3.02) and dogs with lymphoma NRG (43.10% ± 10.24 vs. 57.00% ± 10.31). No significant difference was found between the percentage of MHCII− and MHCII+ monocytes in glucocorticoids-treated dogs with lymphoma (RG) (45.50% ± 16.26 vs. 54.50% ± 16.27). It was also found that the percentage of MHCII− monocytes significantly increased in both groups of dogs with lymphoma (NRG and RG) in comparison to healthy dogs. These results were also shown as the ratio of MHCII+ to MHCII− monocyte percentages, that was significantly lower in both groups of dogs with lymphoma (NRG 1.44 ± 0.57; RG 1.43 ± 0.74), compared to healthy individuals (4.28 ± 0.88) (Figure 3B). The number of CD14+ cells and their subsets (MHCII+ to MHCII−) was calculated based on their percentage in relation to all leukocytes (CD45+ cells) and white blood cell count (cells per microliter of peripheral blood). The number of all CD14+ monocytes was significantly elevated in dogs with lymphoma RG (1781 ± 709.9) when compared to NRG dogs (966.3 ± 551.7) and healthy individuals (812.2 ± 346.2). However, there were no significant differences between the number of monocytes in dogs with lymphoma NRG and healthy individuals (Figure 4A). No significant differences were found in the number of MHCII+ monocytes among groups of dogs (healthy dogs 655.1 ± 280.9; NRG 531.1 ± 283; RG 1013 ± 488.8) (Figure 4B). The number of MHCII− monocytes was higher in dogs with lymphoma RG (768.2 ± 321.1) compared to healthy dogs (157.1 ± 70.27), but there was no significant difference in the number of MHCII− monocytes between dogs with lymphoma NRG (435.8 ± 297.2) and other groups (Figure 4C). A separate chart shows the numbers of all evaluated cell populations in individual patients: MHCII− and MHCII+ monocytes as well as all CD14+ cell populations (Figure 4D).

## 4. Discussion

The study has shown that in healthy dogs only a small population of CD14+ monocytes lacks the expression of MHCII, whereas in dogs with lymphoma (both NRG and RG) their proportion in relation to MHCII+ monocytes increases. This might be associated with an increase in the number of MHCII− monocytes regardless of the treatment or a decrease in the number of MHCII+ cells. However, the number of all CD14+ monocytes in NRG dogs did not differ significantly from the value in healthy animals. It can be assumed that the ratio of MHCII+ to MHCII− monocyte percentages decreases as a result of the loss of MHCII on monocytes, while the total number of CD14+ cells in the peripheral blood does not change. Nevertheless, the reason for the change in the percentage ratio between these two populations in NRG dogs is not clear, especially because in these patients, the numbers of MHCII− and MHCII+ monocytes were not significantly different from healthy controls. Interestingly, there is a clear upward trend in the number of MHCII− monocytes in dogs with lymphoma NRG (as shown in the Figure 4D) despite differences among patients. It should be emphasized that the number of monocytes in dogs may depend on both age, sex and castration [22]. All groups examined in our study were heterogeneous, mainly because canine lymphoma often occurs regardless of age or sex. The number of CD14+ monocytes in each dog depended on the total number of white blood cells and reference values for this parameter in such a polymorphic species is quite wide (6–16.5 × 10^9^ cells/L). Therefore, the number of cells within both monocyte populations was different between individual patients within each group. 

A significant increase in the number of all CD14+ monocytes and MHCII− population was reported only in dogs with lymphoma RG. Glucocorticoids are known to increase the number of monocytes in dogs by releasing the blood marginal pool. It can be concluded that both the increase in the percentage of MHCII− cells in cases of lymphoma and the release of monocytes from the marginal pool due to the action of glucocorticoids contributed to the increase in the number of MHCII− monocytes in dogs with lymphoma RG. Statistical analysis, however, did not show a significant difference in the number of MHCII− monocytes between dogs with lymphoma RG and NRG. This might have been influenced by a varying treatment plan in dogs with lymphoma RG (different glucocorticoids and/or different doses, recommended by clinicians, not the authors). Variations within the group of dogs with lymphoma RG is admittedly a limitation of our study. However, it should be emphasized that collecting a large group of dogs with lymphoma, homogenous in many aspects, is a challenging task. Thus, we decided to include all dogs RG to the experiment and regardless of different treatment protocols, we still made interesting observations. We suggest that in dogs with lymphoma RG, in which the proportion between MHCII+ to MHCII− was decreased, glucocorticoid-dependent release of monocytes from marginal pool allowed us to detect a significant increase in the number of MHCII− cells. A similar conclusion can be made regarding MHCII+ monocytes. Glucocorticoid-dependent release of a reduced percentage of MHCII+ monocytes from the marginal pool to the circulation eliminates the potential difference in the number of monocytes between the groups of dogs with lymphoma RG and healthy animals. This might be an explanation for the lack of a significant difference in the number of MHCII+ cells between these groups of dogs. Otherwise, with significantly reduced percentage of cells, decrease number in the blood should also be expected. 

Interestingly, the decreased ratio between MHCII+ and MHCII− monocyte percentages did not significantly affect the number of both monocyte populations in the peripheral blood of dogs with lymphoma NRG compared to healthy animals. However, it should be emphasized that the changes in the percentage of phenotypic variants of cells is of a greater diagnostic and immunological importance than their number. Thus, the main conclusion from our study is that changes in the proportion between MHCII+ and MHCII− monocytes in dogs with lymphoma have significant impact on the number of circulating monocytes lacking MHCII expression after glucocorticoid administration. However, the experiment did not explain whether glucocorticoids alone influence MHCII+ and MHCII− monocyte percentages, as there was no group of healthy dogs treated with these drugs. On the other hand, there was no difference in the ratio between MHCII+ and MHCII− monocyte percentages comparing two examined groups of dogs with lymphoma (NRG and RG). It may be postulated that the development of the disease is responsible for the changes in MHCII expression rather than glucocorticoids. Little is known about the effects of glucocorticoids and other immunosuppressive agents on monocytes. Kim et al., have found that in human patients with systemic inflammatory response syndrome, HLA-DR is downregulated on both CD14high and CD14low monocytes, which negatively correlates with cortisol levels [23]. This was also confirmed in vitro after incubation of cells from healthy donors with hydrocortisone. Therefore, the influence of glucocorticoids on the expression of MHCII in dogs cannot be excluded until appropriate studies are performed.

The obtained results encourage further research to find the answer to the question regarding the mechanism that is responsible for changes in MHCII expression. Some explanation can be found based on published research, which leads to another hypothesis. The transport of MHCII to the membrane surface of antigen-presenting cells, including monocytes, is a complex process influenced by many factors [24]. Effective antigen presentation and MHCII expression is regulated by cytokines, e.g., IFN-γ, which contributes to the increase of MHCII expression in monocytes in inflammation [25,26]. Therefore, a downregulation of MHCII might be influenced by a factor that interferes with the activity or release of this cytokine. IFN-γ also stimulates the expression of MHC class I molecules on antigen-presenting cells, which increases tumor immunogenicity and stimulate the activity of cytotoxic T lymphocytes [27]. These and other benefits associated with the effects of IFN-γ are used in the treatment of many types of human malignancies using the recombinant form of this protein [28]. The role of IFN-γ in the pathogenesis of canine diseases, including lymphoma, is not clearly understood [29]. Calvalido et al. reported that in dogs with B and T cell lymphoma, serum levels of IFN-γ did not change significantly when compared to healthy individuals [30]. However, the authors have found an increase in the serum concentration of IL-10 in dogs with lymphoma, which was the highest in B-cell type. Interestingly, IL-10 has been shown to decrease the expression of MHCII on the surface of monocytes and impair their ability to present antigen, because of MARCH1 ubiquitination [31,32,33]. Downregulation of MHCII expression on monocytes due to the action of IL-10 also leads to an inhibition of antigen-dependent T cell proliferation [34]. Therefore, it can be hypothesized that an increase in IL-10 serum concentration contributes to the reduction of MHCII expression on monocytes in dogs with lymphoma. An increase in the serum concentration of IL-10 has been observed in humans [35,36]. In patients with NHL, the serum level of this protein is much higher compared to healthy controls and during remission. IL-10 is also considered as a prognostic factor in NHL patients [37,38]. This cytokine is released by malignant B-cells, although in humans it is associated with human immunodeficiency virus or Epstein–Barr virus infections and acquired immunosuppression [39,40,41]. There is no evidence that IL-10 is released by tumor cells in canine lymphoma, but this may be one of the possible causes of negative regulation of MHCII on monocytes. The mechanism that leads to downregulation of MHCII on monocytes in canine lymphoma remains to be elucidated, although it can be assumed that it is significantly related with the immunosuppression and evading immunosurveillance by tumor cells. Interestingly, CD11b+CD14+MHCII− monocytes isolated from the peripheral blood of a dog with osteosarcoma were able to inhibit T cell proliferation, showing immunosuppressive activity [42].

The main limitation of this study was our inability to conduct a comparative analysis between lymphoma types. Difficulties in the collection of many patients with lymphoma prompted us to include all cases whether it was a patient with T- or B-cell lymphoma. The proportion between MHCII+ and MHCII− monocytes in patients with T-cell lymphoma from both groups (NRG and RG) is comparable to those observed in dogs with B-cell lymphoma. However, there were not enough T-cell lymphoma cases to perform a meaningful comparative analysis. Most cases were B-cell lymphomas—80% of dogs NRG and 75% of dogs RG. In both groups, DLBCL lymphoma was the most frequent (70% and 50%, respectively). Moreover, all patients were at an advanced stage of disease (III–V). It can therefore be concluded that the groups were relatively homogeneous. In fact, each case differs in many aspects, which is why we believe they should be considered individually, especially in the field of clinical and laboratory diagnostics. We would also like to emphasize that the aim of the study was not to show differences between histological or phenotypic variants of canine lymphoma. However, it cannot be ruled out that the ratio between MHCII+ and MHCII− monocyte percentages depends, as in humans, on the clinical stage or the lymphoma subtype. Nevertheless, our study shows significant differences that should be considered and better explained in further studies.

## 5. Conclusions

In conclusion, we found that in dogs with lymphoma, regardless of treatment with glucocorticoids, the ratio between MHCII+ and MHCII− monocyte percentages is decreased. While in healthy dogs only a small population of CD14+ monocytes lacks the expression of MHCII, in dogs with lymphoma prior to any treatment and in dogs with lymphoma treated with glucocorticoids, the proportion of MHCII− in relation to MHCII+ monocytes is increased. This finding is like those observed in human lymphoma. Interestingly, we also found that the number of MHCII− monocytes was significantly increased only in dogs treated with glucocorticoids. The resulting data seemed to suggest that this might be due to the release of these cells from the blood marginal pool. Glucocorticoid-dependent release of monocytes from the marginal pool influences the number of both monocyte populations, MHCII− and MHCII+. Further studies are needed to investigate whether monocytes deprived of MHCII protein are associated with impaired immunity in canine lymphoma and what mechanisms are responsible for changes in the expression of MHCII in canine lymphoma.

## Data Availability

The data presented in this study are available on request from the corresponding author.

## Supplementary Materials

The following supporting information can be downloaded at: https://www.mdpi.com/article/10.3390/ani12162135/s1, Table S1: Clinical databases for glucocorticosteroid (GC) administration in the dogs.
