# Supplementary material for: MHCII Expression on Peripheral Blood Monocytes in Canine Lymphoma: An Impact of Glucocorticoids
Source: Animals (Basel). 2022 Aug 19;12(16):2135. doi: 10.3390/ani12162135 (PMC9404857; doi:10.3390/ani12162135)
Supplement: Supplementary file 1 [file animals-12-02135-s001.zip › animals-1789773-supplementary.pdf]

Table 1. Clinical databases for glucocorticosteroid (GC) administration in the dogs

Breed	GC	Period of GC Administration before Blood Collection
mixed breed	dexamethasone	4th, 3rd, 2nd day
mixed breed	prednisone	7 days
mixed breed	dexamethasone	5th day
mixed breed	dexamethasone / prednisone	* < 10th day and 5 days
mixed breed	prednisone	3 days
Rottweiler	methylprednisolone	7th, 6th, 5th days
German Shepherd	dexamethasone	8th and 5th days
English Cocker Spaniel	dexamethasone	* < 1st day

* these dogs have been receiving GC before these days but no specific data about administration schedule are known
